# Overview of the Zinc Functional Interactome Through Health Hallmarks and Medical Conditions

**DOI:** 10.3390/nu18020336

**Published:** 2026-01-21

**Authors:** Mirela Pavić Vulinović, Vedran Micek, Davorka Breljak, Ivana Vrhovac Madunić, Josip Madunić, Marija Ljubojević

**Affiliations:** 1 Department of Anatomy, Histology and Embryology, Faculty of Veterinary Medicine, University of Zagreb, 10000 Zagreb, Croatia; mpavic@vef.unizg.hr; 2 Animal Breeding Unit, Institute for Medical Research and Occupational Health, 10000 Zagreb, Croatia; vmicek@imi.hr; 3 Division of Toxicology, Institute for Medical Research and Occupational Health, 10000 Zagreb, Croatia

**Keywords:** aging, amino acids, essential, immunomodulation, inflammation, minerals, neurodegenerative diseases, neurodevelopmental disorders, nutrients, vitamins

## Abstract

Zinc is an essential micronutrient involved in structural, catalytic, and regulatory functions across all levels of biological organization. Despite substantial advances over the past two decades, the zinc literature remains highly fragmented, with mechanistic, nutritional, and clinical findings often reported in isolation. Additionally, the synergistic interactions between zinc and other micronutrients—particularly minerals and vitamins—are dispersed across multiple research domains, complicating efforts to understand their integrated roles in maintaining homeostasis. Recent developments in artificial intelligence (AI) present new opportunities to consolidate these data, enabling multi-scale analyses of zinc-dependent processes and the broader zinc interactome. Although a complete map of the zinc interactome is not yet feasible, an integrative perspective is needed to contextualize zinc’s contributions within the framework of the hallmarks of health. This narrative review highlights zinc’s involvement in cellular maintenance, metabolic regulation, stress response, and systemic physiological function. It further examines how disruptions in zinc status, alone or in combination with other nutrient imbalances, contribute to clinically relevant disorders. By combining current knowledge across molecular, cellular, and systems biology levels, this review illustrates zinc’s pleiotropic effects on physiological resilience and healthspan, with particular emphasis on its role in nutritional status, homeostatic regulation, and overall human health.

## 1. Introduction

Zinc (Zn) performs essential and multifaceted functions in human physiology, yet its full biological significance remains widely underestimated. Evidence accumulated in the 21st century demonstrates, through numerous representative examples, that zinc contributes to every level of biological organization—from ionic interactions and molecular signaling to organelle dynamics, cellular function, tissue integrity, and systemic homeostasis. Approximately 10% of human proteins bind biologically active zinc ions (Zn^2+^) influencing a broad spectrum of catalytic, structural, and regulatory processes, as illustrated through examples in this hypothesis-generating view of zinc’s potential role as a central coordinating element within the system biology concept of the whole interactome [1,2,3,4].

In this review, we examine examples of zinc’s relevance through three major hallmarks of health and emphasize that zinc homeostasis is defined not only by total body content but by spatial compartmentalization, dynamic buffering, and tightly regulated intracellular distribution. Zinc functions both as a transient signaling ion and as a catalytic and structural component of proteins, maintaining genomic stability, supporting adaptive stress responses, and contributing to metabolic balance. Although not all health or aging hallmarks are directly zinc-dependent, zinc deficiency—especially when accompanied by insufficient levels of synergistic nutrients—can compromise interconnected regulatory pathways and thereby open new avenues for interventions aimed at enhancing healthspan and resilience [5,6,7].

Extensive prior research, including foundational biochemical and physiological analyses, has characterized zinc’s chemical properties, storage forms, transport systems, signaling mechanisms, and proteome-wide interactions. Emerging computational tools, including artificial intelligence models such as AlphaFill integrated with AlphaFold, now enable more accurate reconstruction of zinc-binding sites and zinc-mediated protein–protein interactions (ZPPI). These innovations create new opportunities to link zinc biochemistry with higher-order biological processes and to develop comprehensive models of zinc-dependent system regulation [1,2,3,4,7,8,9].

However, fully integrating zinc biology across molecular, cellular, and organismal scales remains a substantial challenge due to the structural complexity of the zinc interactome throughout the inherently open, adaptive nature of living systems. A systems-biology framework is therefore necessary to contextualize examples of zinc’s functions within health, aging, and disease. This positively inclined overview refers to the relatively small number of recent, comprehensive and critical reviews published in the PubMed database to combine elements of zinc biology—chemical characteristics, evolutionary relevance, transport and signaling pathways, essential proteome interactions, nutrient synergies, and (patho)physiological consequences—to illustrate how zinc supports whole-organism function in the processes that underlay health adaptable targets with examples of established backgrounds in certain Zn-related disorders.

Although contemporary tools cannot yet resolve the full multilayered complexity of the zinc interactome, the gathered ideas, experience, knowledge and information through this first quarter of 21st century open possibility for conceptual exploratory framework presented here. Improved understanding of zinc’s integrative roles across biological scales may inform future research into nutritional strategies, directed adjuvant therapy, and support investigations into the maintenance of physiological resilience throughout the lifespan.

Without AI-driven approaches, constructing such multidimensional biological integration would be exceedingly difficult. When viewed against the many unresolved questions in zinc biology, the present review represents only an initial outline chosen out as positively examples of the vast complexity that remains to be fully mapped. Ultimately, the central question becomes whether we should effectively “galvanize” ourselves to avoid the metaphorical “rust” of premature functional decline—an effort that may require future integrative researching, recognizing and leveraging the foundational contributions of zinc.

## 2. Comprehension of Zinc Interactome

The triage theory advanced by Ames posits that even marginal insufficiencies in essential nutrients redirect metabolic resources toward immediate survival needs, thereby downregulating physiological processes associated with long-term health, repair, and longevity [7]. Within this conceptual framework, each hallmark of health is contingent upon the adequate supply, bioavailability, and functional utilization of essential and conditionally essential nutrients, including Zn. In contrast, aging—defined as the gradual loss of defined hallmarks of health—and numerous chronic diseases are seldom attributable to nutrient excess. Rather in the case of Zn, these states more commonly arise from deficiency, dysregulation, or mislocalization within cellular and systemic compartments [4,5,6,7,8,9].

Zinc is an essential trace element with distinctively broad functional involvement across structural, catalytic, regulatory, and signaling pathways. Its biologically active forms—including tightly bound structural zinc, transiently exchangeable zinc pools, and dynamic zinc signals—participate in virtually all hallmarks of health adaptable factors. The concept of a zinc functional interactome integrates these roles by describing zinc as a central coordinating node linking epigenetic regulation, transcriptional control, enzyme activity, immune competence, redox homeostasis, endocrine balance, and cellular renewal processes. Despite Zn’s dominant involvement in these processes, many molecular and mechanistic reviews fail to adequately emphasize the dependence of these pathways on essential nutrients. Observing health through the positively inclined lens of Zn-dependent molecular processes, as undertaken in this review, underscores Zn’s important roles in maintaining cellular metabolism, enzymatic architecture and function, genome stability, oxidative balance, and immune competence. Disruption of Zn homeostasis—whether through insufficient intake, impaired absorption, excessive losses, or intracellular misdistribution—partially contributes to metabolic imbalance, accelerates aging-associated decline, and participates in the etiology of inherited and acquired pathologies [5,6,7,8,9,10,11,12,13,14,15,16].

There remains a critical need for enhanced awareness among researchers, clinicians, and public health stakeholders regarding the growing body of evidence linking micronutrient status to molecular and physiological resilience. When health is viewed solely through overarching frameworks such as the hallmarks of health, aging paradigms, or lifestyle-based models (e.g., “old NEWS”: Nutrition, Exercise, Weight control, Sleep, and Social interaction versus Stress), molecular and biochemical perspectives are often insufficiently integrated. In particular, mutual synergy even of ions and small molecules—central components of signaling pathways, enzymatic systems, and metabolic networks—are frequently overlooked in interactome-based analyses [8,15,16,17].

**Figure 1:**
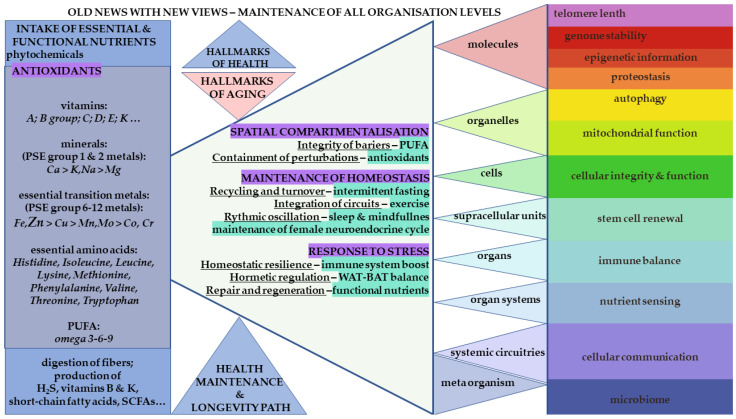
Schematic representation of the integrated nutrient–zinc interactome and its relationship to the hallmarks of health. Essential and functional molecules, including zinc (Zn; enlarged at left), are depicted alongside other micronutrients and macronutrients required to sustain physiological integrity. These elements are shown within blue rectangles on the left, emphasizing their dependence on adequate hydration and their collective contribution to cellular nutrition, metabolic regulation, and organismal homeostasis. The central section illustrates the hallmarks of health, accompanied by selected strategies for their maintenance. Further, the right-side highlights adaptable factors for function, regulatory pathways, including microbiome. Imbalances of these malleable targets are associated with aging processes and a loss of homeostatic control. The horizontal triangular layers represent the hierarchical organization of the human body—from molecular and cellular processes to tissues, organs, and integrated physiological systems—each of which requires sufficient nutrient availability to function optimally. The twelve targeted physiological processes or characteristics depicted in the right-hand rectangles illustrate domains that are directly or indirectly dependent on appropriate nutrient status, including Zn homeostasis. The gastrointestinal (GIT) microbiome is positioned as a meta-organism with bidirectional interactions with host nutrient status, with selected functional roles indicated in the lower left portion of the scheme. Abbreviations: NEWS—Nutrition, Exercise, Weight control, Sleep, and Social interactions versus Stress; WAT—white adipose tissue, functioning primarily as an energy-storage compartment; BAT—beige adipose tissue, a mitochondria-rich adipose subtype involved in thermogenesis and cold adaptation. This figure integrates established concepts of the hallmarks of health within a dynamic, open-system perspective, underlining the malleability and resilience conferred by sufficient and balanced intake of essential nutrients [5,6,7].

In this context, Zn is positioned in this review as a primary element among the essential transition metals (eTMs). The broader interactome is conceptualized as a dynamic network comprising metals, amino acids, metabolites, polyunsaturated fatty acids (PUFAs), vitamins, antioxidants, and phytonutrients that collectively sustain human physiology (Figure 1) [7]. The objective is to illustrate, through selected examples, how the Zn proteome interacts synergistically with small molecules and other proteins to support integrated cellular and systemic function.

Zn operates in synergy with vitamin D_3_ (VD) and close coordination with minerals such as calcium (Ca^2+^) and magnesium (Mg^2+^) in immune regulation, neuromodulation, metabolic homeostasis, and intracellular signaling [2,7,8,9,10,11,12,13,14,15,16,17,18,19]. Evolutionary evidence from bioinformatic analyses further supports the fundamental role of Zn in biological systems. The conservation of established domains across the phylogenetic tree, together with their proposed involvement in prebiotic chemistry, supports hypotheses such as the “zinc world,” which posit Zn as a core determinant of early biochemical evolution [20,21].

At the molecular level, zinc is indispensable for the structural integrity of zinc-finger transcription factors, nuclear receptors, epigenetic writers and erasers, and chromatin-remodeling complexes. Exchangeable zinc modulates signaling cascades, including kinase and phosphatase networks, and influences Ca^2+^-dependent neurotransmission through inhibition or modulation of glutamatergic receptors. Zinc also supports proteostasis through lysosomal acidification, autophagic flux, and metallothionein-mediated detoxification and antioxidant defense.

Through these mechanisms, zinc contributes to the maintenance of major adaptable factors within hallmarks of health, including genomic stability, proteome maintenance, efficient immune responses, balanced inflammatory signaling, metabolic homeostasis, and cellular resilience to stress. Deficiency or mislocalization of zinc disrupts these processes and is implicated in wide-ranging medical conditions. These include cardiometabolic and renal disorders, immune dysfunction and chronic inflammation, neurodegeneration, neurodevelopmental disorders including autism spectrum disorder (ASD) and Kleefstra syndrome, endocrine disturbances such as those associated with menopause transition, and age-related decline. In specialized contexts such as Wilson’s disease, zinc therapy demonstrates a well-defined mechanistic rationale and established clinical efficacy.

Taken together, the examples presented within the Zn functional interactome provide a framework linking micronutrient sufficiency to systemic regulation across the lifespan. Understanding Zn’s coordinated molecular roles offers translational insights that may inform preventive research and supportive strategies for both common and rare diseases.

## 3. Properties of Essential Transition Metals in the Organic World

### 3.1. Place of Essential Transition Metals, eTMs in Periodic Table, Life and Evolution

Historically, the Renaissance physician and German “father of pharmacology and toxicology,” Paracelsus (1493–1541), was responsible for assigning the name to the metal zinc (Zn) [19]. Paracelsus is more widely recognized for articulating the foundational principle of toxicology: “All substances are poisons; there is none that is not. The dose differentiates a poison from a remedy.” This principle is universally applicable, encompassing not only essential micronutrients such as Zn but even the intake of water.

In biological systems, iron (Fe), Zn, and the lower-abundance copper (Cu) form a triad of interlinked and mutually dependent essential metals. Manganese (Mn) and cobalt (Co), although required in smaller quantities, also contribute to specific cooperative biochemical functions. Zn, being chemically relatively inert, does not participate in redox cycling, in contrast to Fe and Cu. This characteristic reflects its electronic configuration—namely, a full 3d orbital and the loss of two 4s electrons—stabilizing the Zn^2+^ oxidation state [1]. Nevertheless, Zn exhibits redox responsiveness at the biological level due to its dynamic coordination with amino acids, most notably sulfhydryl-containing cysteine (Cys) residues and the imidazole group of histidine (His). Toxic metals from the same group, including cadmium (Cd) and mercury (Hg), exhibit strong affinity for these same residues and can displace Zn from its physiological binding sites, resulting in mislocalization, dysregulation, and the initiation of cellular damage pathways [1,2,9,19,20,21,22].

From an evolutionary standpoint, Zn and Fe metabolisms appear to reflect longstanding interactions among eTMs acting as mediators and catalysts during the emergence and diversification of early biochemical systems. The widely discussed “zinc world” hypothesis, supported by the work of Mulkidjanian, posits that early Earth environments were enriched in Zn/ZnS nanoparticles, in contrast to Russell’s FeS/FeS_2_-based hydrothermal model [20,21]. Following the formation of the Moon during the “Giant Impact,” differential evaporation and metal settling contributed to the establishment of Earth’s Fe–Ni–rich core, while the more volatile Zn accumulated nearer the surface. This geochemical distribution has been proposed as an explanation for Zn’s persistent prevalence in biochemical systems supporting the evolution of complex life.

Zinc did not replace iron but functioned in parallel as early life forms expanded in biochemical complexity, eventually giving rise to diverse archaeal, bacterial, and eukaryotic lineages. In contemporary organisms, the Zn interactome is maintained by coordinated compartmentalization through transport, storage, and buffering via an extensive array of proteins. Zn further protects against Fe-mediated reactive oxygen species (ROS) generation and associated oxidative damage through both direct mechanisms (e.g., metal displacement reactions) and indirect mechanisms (e.g., Zn-dependent signaling and enzyme activation) that contribute to stress adaptation and cellular resilience.

Proteins utilize Zn through multiple mechanisms, including participation in zinc-dependent protein–protein interactions (ZPPIs), regulation of post-translational modifications (PTMs), and control of transcriptional, translational, and epigenetic processes. Through these diverse functions, Zn plays a principal role in maintaining organismal homeostasis. Collectively, these interactions constitute a complex and dynamic zinc interactome that interfaces with other essential nutrients and cofactors, forming an integrated network for balanced biological function and overall health. The subsequent sections of this review elaborate on examples of these mechanisms, highlighting established interactions across multiple organizational levels within the organism [1,2,8,19,20,21,22,23,24,25,26,27,28,29,30,31,32,33].

### 3.2. Mitochondrial Challenges, Spatial Compartmentalization and Homeostasis Maintenance

In aerobic organisms, iron plays a paradoxical yet indispensable role: it is required for the delivery and storage of oxygen, functioning as a central component of hemoglobin and myoglobin. Within mitochondria—the endosymbiotic organelles essential for cellular respiration—divalent Fe participates in redox cycling that underlies oxidative phosphorylation and ATP generation through the metabolism of carbohydrates. The evolutionary transition from unicellular anaerobic microbes to oxygen-utilizing eukaryotic cells was facilitated by spatial compartmentalization, including the incorporation of mitochondria and the organization of their internal membranes.

However, mitochondrial activity inevitably produces ROS, primarily superoxide radicals generated at the electron transport chain (ETC) located on the inner mitochondrial membrane (IMM). These radicals are rapidly detoxified by manganese-dependent mitochondrial superoxide dismutase (Mn-SOD, SOD2). In parallel, cytosolic and extracellular Cu/Zn-dependent superoxide dismutase (SOD1 and SOD3, respectively) catalyze similar conversions in different cellular compartments. Collectively, these enzymes convert superoxide to hydrogen peroxide (H_2_O_2_), a less reactive intermediate. If not further metabolized, H_2_O_2_ can participate in Fenton chemistry with Fe, generating highly damaging hydroxyl radicals. Catalase (CAT), an Fe-heme–containing enzyme, efficiently degrades H_2_O_2_, preventing this escalation of oxidative stress [1,2,19,22,23]. This sequence of reactions illustrates the essential cooperation among multiple eTMs and emphasizes the importance of compartmentalization and stress-response regulation, with ROS functioning not only as damaging agents but also as examples of hormetic signaling molecules within hallmarks of health adaptable factors (Figure 1).

Efficient ETC activity requires a sufficient supply of reducing equivalents, NADH and FADH_2_, produced by the tricarboxylic acid (TCA) cycle within the mitochondrial matrix. Zinc stored in the intermembrane space (IMS) can modulate this metabolic flux by inhibiting α-ketoglutarate dehydrogenase, a key enzyme responsible for NADH production and proton release, with carbon dioxide (CO_2_) as a metabolic by-product [23]. Carbonic anhydrases (CAs), among the most evolutionarily conserved and rapid Zn-dependent enzymes, subsequently catalyze the hydration of CO_2_ to carbonic acid. This reaction facilitates the formation of bicarbonate (HCO_3_^−^), which is transported in exchange for chloride ions to maintain systemic pH homeostasis. Approximately 80% of circulating CO_2_ is transported in this bicarbonate form, contributing to acid–base buffering and ultimately being reconverted to CO_2_ for exhalation [23,24].

The proper functioning of mitochondria as the cellular “powerhouse” is therefore central to maintaining whole-organism homeostasis. The regulatory pool of Zn within the IMS reflects its role in modulating energy production. As eukaryotes evolved increasing multicellular complexity, the coordination of Zn-, Fe-, Cu-, and Mn-dependent processes became essential for cellular specialization, circulatory integration, maintenance of barrier integrity, and diversification of cellular phenotypes. These processes rely on the capacity of a single genome to generate numerous functional outputs through epigenetic regulation and imprinting—mechanisms that will be addressed in subsequent sections within the chosen positive instances of the zinc interactome broader context.

### 3.3. Character of Essential Transition Metals, eTMs and Response to Intracellular Stress

The biological functions of the eTMs triad—iron, zinc and copper—as well as those of all exogenously ingested compounds, including nutrients and their metabolites, are governed by the principles of ADMETox, encompassing absorption, distribution, metabolism, excretion, and toxicity. These metals undergo tightly regulated intestinal absorption, controlled distribution within specific biochemical environments, feedback-regulated metabolic processing or binding to functional molecules, storage in appropriate protein complexes—with or without dedicated compartmentalization—and eventual conversion to excretable forms. Despite these regulatory mechanisms, eTMs retain the potential for bioaccumulation and toxicity under conditions of dysregulation.

Among the eTM triad, Fe is the most hazardous due to its capacity for Fenton reactivity. Fe has no physiological elimination pathway and progressively accumulates within cells of various organs over the lifespan. Excess Fe is sequestered primarily in ferritin nanocage complexes or aggregates as hemosiderin within lysosomes and the cytosol, where its bioavailability can perpetuate ROS generation [1,9,22]. Catabolism of Fe-rich ferritin or damaged mitochondria proceeds via ferritinophagy and mitophagy, respectively, within the endosomal–autophagolysosomal pathway (EALP). Lysosomal acidification liberates reactive Fe, which drives oxidative stress and may further destabilize cellular homeostasis.

Zn counteracts Fe-mediated oxidative injury both indirectly—through its involvement in antioxidant enzymes and stress-responsive proteins—and directly, through its function as a released signaling ion. Increases in intracellular free Zn activate the metal-responsive transcription factor (MTF1), which binds metal response elements (MREs) and modulates the expression of numerous protective proteins, including inducible metallothioneins (iMTs) [2,19,22,26,27]. Persistent redox stress activates additional protective mechanisms, including Cys oxidation and the release of Zn from KEAP1 sensor motifs, resulting in the stabilization and nuclear translocation of Nrf2. Nrf2 subsequently binds antioxidant response elements (AREs) within gene promoters and induces transcription of more than 200 cytoprotective proteins, including ferritin and iMTs for the sequestration of Fe and Zn, respectively [12].

When oxidative stress extends to DNA damage, Zn-dependent poly(ADP-ribose) polymerases (PARPs) mediate genome surveillance by coordinating DNA repair through PTMs and recruitment of DNA repair complexes [28]. Collectively, these mechanisms illustrate how eTMs—and particularly Zn—participate in adaptive responses to exogenous or endogenous stressors, reinforcing homeostatic resilience. These interactions span multiple levels of organization, from metal-binding proteins to organelle degradation pathways, including autophagy and lysosomal recycling. Altogether, these processes integrate directly with several hallmarks of health adaptable factors, including mitochondrial function, proteostasis, autophagy, and genome stability (Figure 1).

Fe and Zn are also evolutionarily and functionally linked to Cu, the third member of the eTM triad. Although present in tissues at lower concentrations, Cu is essential for redox transitions of Fe during transport, storage, and enzymatic reactions [1,9,22,23]. Disruption of the Zn–Cu–Fe axis contributes to redox imbalance and increases susceptibility to degenerative and metabolic diseases associated with aging [8,9,22,25].

Under specific stress conditions, all three eTMs can participate in distinct forms of regulated cell death. Zn can promote apoptosis when released from the endoplasmic reticulum or mitochondria, inhibiting metabolic enzymes and mitochondrial respiration. Fe drives ferroptosis, a form of cell death characterized by iron-dependent lipid peroxidation, primarily of long chain PUFAs, under conditions of cysteine depletion, which is essential for the synthesis of the major cellular protective tripeptide glutathione (GSH). Excess Cu triggers cuproptosis, a recently defined mechanism of regulated cell death in which Cu induces aggregation and loss of function of mitochondrial lipoylated enzymes, particularly components of the pyruvate dehydrogenase complex (PDC) within the TCA cycle [1,2,8,9,19,22,23,24,25].

These interconnected examples underscore the centrality of eTMs—particularly Zn metal–protein interactions—across multiple hallmarks of health encompassing regulated cell death, mitochondrial homeostasis, stress resilience, and genome protection as integral malleable targets (Figure 1).

## 4. Storage, Transport and Signaling of Zinc

### 4.1. Zinc Storage

ZNG1, recently identified as a Zn-specific escort protein, facilitates the targeted delivery of Zn^2+^ to methionine aminopeptidase 1 (METAP1), an enzyme for processing protein *N*-terminal methionine (Met) removal on cytosolic protein. This discovery positions ZNG1 within the broader group of Zn-storage and Zn-trafficking proteins, complementing the long-recognized roles of inducible and constitutive metallothioneins (MTs). MTs are evolutionarily conserved, cysteine-rich proteins that coordinate the binding of up to seven Zn ions through their α- and β-domains. Although MTs are not essential for survival, they are crucial for Zn buffering, intracellular metal distribution, and protection against oxidative and electrophilic stress. Their expression can be induced by Zn itself, glucocorticoids, inflammatory mediators, and diverse environmental stressors [2,8,19,22,26,27].

Experimental animal studies have demonstrated that overexpression of inducible MTs (iMTs) enhances stress resistance, improves systemic health parameters, and increases lifespan in mice by approximately 30%. Notably, comparative analyses in wild-type male and female mice revealed that Zn supplementation alone is sufficient to extend lifespan in both sexes, independent of iMTs overexpression, underscoring the possible contribution of Zn homeostasis to organismal longevity [2,22,27].

Human genetic data further support the physiological significance of MTs in aging. Population studies showed that female centenarians frequently exhibit MT1A polymorphisms that increase Zn-binding affinity, particularly within the α-domain containing 11 cysteine residues. This enhanced affinity may stabilize Zn sequestration while sustaining physiologically accessible Zn pools for controlled release and utilization in high-demand processes. MT1A longevity variants may enhance Zn availability for tissues with rapid turnover, including erythropoietic lineages in the bone marrow. Adequate Zn status is needed for red blood cell maturation, immune cell development, and bone remodeling, all of which could contribute to healthy aging trajectories, and deserve further studies [25,29,30,31].

It is important to note that MT concentrations in serum or circulating blood cells are not reliable biomarkers of systemic Zn status. MTs may exist in metal-bound or metal-free (thionein) states, participate in redox buffering, and exert additional regulatory functions that remain incompletely characterized [8,19]. The combination of Zn sequestration, controlled release, and strict spatial compartmentalization—coordinated by MTs, ZNG1, and other Zn-trafficking proteins—represents a mechanism by which Zn is safely stored, mobilized, and utilized across a wide physiologically tolerated range and intracellular concentrations.

### 4.2. Zinc Transport and Signaling

As noted above, the intracellular and extracellular concentrations of eTMs must be tightly regulated to prevent toxicity and maintain homeostasis. In accordance with this requirement, zinc transport is governed by two major protein families: the ZIP (Zrt-/Irt-like Protein) transporters, belonging to the solute carrier SLC39A family, and the ZnT (Zinc Transporter) proteins, belonging to the SLC30A family. Initially identified in plants for their ability to transport divalent iron, the fourteen mammalian ZIP transporters mediate Zn influx into the cytosol, either from the extracellular environment or from intracellular organelles. Cytosolic import of divalent metals is further supported by the broadly acting divalent metal transporter 1, DMT1 (SLC11A2), which transports Fe^2+^ but can also carry Mn^2+^ and Zn^2+^ under certain conditions.

In contrast, the ten characterized ZnT transporters facilitate Zn efflux from the cytosol or direct Zn into organelles including the Golgi apparatus, endoplasmic reticulum (ER), and secretory vesicles. Together, these coordinated influx and efflux systems ensure appropriate Zn distribution across organelles and cellular compartments. Both transporter families exhibit partial substrate promiscuity, allowing transport of additional divalent metals such as Fe^2+^ and Mn^2+^. Ongoing research continues to refine our understanding of transporter specificity, regulatory mechanisms, and structural determinants of metal selectivity [2,8,32].

Zn concentrations in the cytosol can be rapidly adjusted through release from intracellular stores such as vesicles, lysosomes, mitochondria, or the ER. Although total Zn content is high (mM range) in these compartments, free cytosolic Zn typically remains in the picomolar–nanomolar range. Transient increases in free Zn^2+^ act as localized intracellular or extracellular signals that modulate enzyme activity, influence signaling cascades, and regulate ZPPIs, even in the presence of far higher concentrations of Ca^2+^ and Mg^2+^ [2,4,19,22,27].

The dynamics of Zn signaling can now be visualized in real time using Zn-specific fluorescent probes in living cells, revealing rapid, localized Zn fluxes involved in diverse physiological processes [1,2,4,8,19]. As emphasized throughout the examples presented in this review, spatial compartmentalization is one of the central hallmarks of healthy Zn physiology. Disruption of Zn transporter localization, expression, or function contributes to altered Zn distribution, impaired buffering, and consequent loss of homeostatic control. Such dysregulation is increasingly recognized as a contributing factor in a broad spectrum of metabolic, inflammatory, neurodegenerative, and age-associated disorders.

## 5. Presence of Zinc in Various Protein Structures and Functions

### 5.1. Zinc in All Enzyme Families

Even prior to the widespread adoption of AlphaFold2 and AlphaFill, bioinformatic analyses had established that zinc participates in all six major enzyme classes. AI-driven structural evaluations further demonstrated that zinc is positioned in catalytic sites approximately three times more frequently than in purely structural roles, with dual catalytic–structural positioning accounting for more than 10% human proteins, and a smaller fraction remaining functionally unresolved. Notably, nearly one-third of zinc-dependent catalytic enzymes exhibit hydrolytic activity, reflecting the broad biochemical importance of Zn across pathways essential for cellular homeostasis. Carbonic anhydrase, historically the first enzyme confirmed to require zinc for activity, now exemplifies a large and phylogenetically diverse family distributed extensively across organisms. CAs are just one already mentioned illustration in concept of Zn interactome that will be asserted through further selected processes within hallmarks of health [1,2,3,4,5,8,19].

### 5.2. Zinc in Enzymes Governing Cellular Identity

Beyond its catalytic functions, zinc is indispensable for a remarkably broad repertoire of Zn-containing proteins involved in PTMs and the multilayered epigenetic mechanisms that preserve differentiated cellular identity [32,33,34,35,36]. Zinc contributes not only to enzymes functioning as “writers” of epigenetic marks, but also to those serving as “readers” and “erasers,” as well as to numerous additional PTM-mediating enzymes integrated within the chromatin regulatory network.

A central example is DNA methyltransferase 1 (DNMT1), a human Zn-dependent enzyme responsible for maintaining repressive epigenetic signals through cytosine methylation (5-methylcytosine, 5mC). Beyond DNA methylation, zinc is required for multiple histone-modifying enzymes that regulate chromatin accessibility and transcriptional outcomes. Methylation and acetylation of lysine (Lys) and arginine (Arg) residues serve as key chromatin marks associated with gene activation or repression. The coordinated regulation of gene expression is therefore mediated by zinc-dependent histone methyltransferases (PKMTs, PRMTs) and demethylases (e.g., KDM2A), as well as histone acetyltransferases (HATs) and deacetylases (HDACs) belonging to classes I, II, and IV, respectively.

Importantly, zinc is also structurally required for the function of the NAD+-dependent sirtuins (class III HDACs/KDACs), a fact that is often overlooked. The seven members of the SIRT family are differentially localized across tissues and organelles, where they play central roles in sustaining cellular identity, genomic stability, and metabolic–epigenetic integration within the broader epigenetic landscape [8,32,33,34,35,36].

DNA methylation and zinc-dependent chromatin-remodeling processes collectively contribute to sex-specific epigenetic signatures established through genomic imprinting. These imprinting mechanisms are essential for mammalian reproduction, embryonic development, and evolutionary adaptation [37]. As illustrated in Figure 1, the presence of zinc within many enzymes central to epigenetic regulation underscores its role as a dynamic and nutritionally responsive modulator of differentiation and chromatin organization within the maintenance of hallmarks of cellular health.

### 5.3. Zinc Finger Proteins

Zinc finger proteins (ZNFs) constitute one of the largest and most functionally diverse classes of zinc-binding proteins and represent a dominant structural motif among zinc-containing enzymes and regulatory factors. These proteins encompass multiple domain architectures with distinct metal-coordination patterns, and their functional repertoire continues to expand. ZNFs participate in a wide array of cellular processes, including the regulation of epigenetic mechanisms and PTMs described above, as well as sequence-specific binding to DNA and RNA, interactions with nuclear receptors such as peroxisome proliferator-activated receptors (PPARs), and association with cytoskeletal components [38,39].

A substantial body of literature has delineated the structural and functional complexity of ZNFs, with several major subclasses receiving particular attention. Among these, the C2H2 zinc finger domain represents the classical motif primarily associated with transcriptional regulation. RING finger domains are central to ubiquitin (Ub) conjugation and part of protein degradation and PTMs pathways, while PHD fingers function in chromatin interpretation and epigenetic modification. LIM domains contribute to cytoskeletal dynamics through interactions with actin-associated proteins. Collectively, these representative subclasses illustrate the breadth of ZNF involvement across transcriptional control, protein turnover, chromatin remodeling, and cytoskeletal organization [38,39].

Overall, ZNFs comprise more than 30 structurally distinct motifs and represent one of largest protein families, integrating zinc into the regulation of the majority of cellular processes [2,8,19,38,39]. The following section provides an overview of selected transcription factors as a subset of ZNFs, highlighting their role in information flow, stress sensing, and position of zinc for lipid-soluble hormones, vitamins, and other metabolic cues.

### 5.4. Zinc-Dependent Transcription Factors

Characteristic zinc finger (ZNF) transcription factors, often referred to as zinc-dependent transcription factors (ZTFs), contain zinc coordinated by two histidine and two cysteine residues (C2H2) within each finger domain that binds to DNA. These proteins play a central role in maintaining chromosomal dynamics, as described previously, with classical examples including the first-identified transcription factor TFIIIA and the metal-responsive transcription factor-1 (MTF-1). ZNFs represent the largest structural class within transcription factors and function as essential regulators of gene expression, exerting both transcriptional activation and repression to control messenger RNA synthesis and the flow of genetic information [2,8,19,38,39].

It is important to emphasize that all nuclear receptors (NRs) are themselves specialized ZTFs. NRs comprise multiple families that bind structurally diverse ligands, including seco-steroid vitamins, steroid hormones, and other endocrine regulators of metabolism. Each NR monomer contains a conserved DNA-binding domain (DBD) composed of two zinc finger motifs, enabling recognition of palindromic hormone-response elements in gene promoters. Functional activity typically requires ligand-dependent homo- or hetero-dimerization. Classical NRs include receptors for neuroendocrine hormones, sex steroids—estrogen receptor (ER), progesterone receptor (PR), and androgen receptor (AR)—as well as adrenal gland–derived hormones glucocorticoid receptor (GR) and mineralocorticoid receptor (MR), which mediate responses to cortisol and aldosterone, respectively. Other essential NR ligands include seco-steroids such as vitamin D binding the vitamin D receptor (VDR), and vitamin A derivatives binding the retinoid X receptor (RXR). Thyroid hormone receptor (THR) binds iodinated thyroid hormones, while the peroxisome proliferator-activated receptors (PPARs) form a broad NR subfamily responsive to fatty acids, prostaglandins, leukotrienes, and several nutrigenomic compounds including curcumin [8,40,41].

These examples illustrate the central role of zinc in the structural integrity and transcriptional function of all NRs. Their actions convey systemic information about metabolic, endocrine, and nutritional status to maintain organismal homeostasis; for example, rhythmic oscillations—identified as a hallmark of health—also depend on appropriate zinc availability (Figure 1) [5]. For instance, diurnal cortisol secretion requires zinc to support metabolic regulation throughout the day and to facilitate rapid glucocorticoid signaling under stress. Similarly, the cyclic fluctuations of neuroendocrine hormones across the menstrual cycle underscore zinc’s essential role in female reproductive physiology and its potential involvement in menopausal transition when deficiency may be present [14].

Beyond classical ZTFs and NRs, additional zinc-dependent transcription factors include the well-characterized tumor suppressor p53 and its homologs p63 and p73. These multi-domain proteins require a single zinc ion coordinated within the DNA-binding domain to maintain proper folding necessary for dimer or tetramer formation. Zinc stabilizes the DBD and is essential for accurate recognition of consensus DNA response elements, structural flexibility, and transcriptional activity. p53 function integrates tightly with zinc-dependent regulatory networks governing cell survival and apoptosis. Under non-stress conditions, p53 is rapidly ubiquitinated and degraded via the ubiquitin–proteasome system (UPS). Components of this pathway—including the E3 ubiquitin ligase MDM2, a major p53 regulator—also contain zinc-binding motifs essential for catalytic function.

The tumor-suppressive role of p53 can often be restored in cancers through zinc supplementation, and recent advances in synthetic zinc metallochaperones (ZMCs) have demonstrated promising therapeutic effects by reactivating multiple p53 mutants [8,38,42]. Within the framework of the hallmarks of health, zinc-dependent regulation of p53 contributes to controlled cell-cycle progression and maintenance of stem cell renewal, while zinc involvement in the UPS integrates directly into proteostasis, ensuring proper cytosolic protein turnover (Figure 1).

## 6. Zinc Nexus with Amino Acids, Vitamins, and Minerals in Health and Disease

### 6.1. Zinc, B Vitamins, and Methionine Metabolism

Beyond its well-established roles in one-carbon (1C) metabolism and epigenetic regulation, zinc is obligatory for the synthesis and maintenance of information-carrying molecules involved in nucleic acid and protein methylation. Mammals rely exclusively on S-adenosylmethionine (SAMe) as a universal methyl-group donor, generated from the essential sulfur-containing amino acid methionine, Met. Critical steps of the Met cycle require zinc-dependent enzymatic activity, particularly of methionine synthase (MS) and betaine–homocysteine methyltransferase (BHMT), which operate in biochemical synergy with folate (vitamin B9) provided as tetrahydrofolate (THF) and vitamin B12 (cobalamin). When Met intake exceeds metabolic capacity, homocysteine (Hyc) accumulates, representing a potentially harmful intermediate unless it is effectively recycled or catabolized [8,34,43,44].

Homocysteine catabolism proceeds through the transsulfuration pathway, generating Cys, which is essential for further protein synthesis and for the production of the major intracellular antioxidant tripeptide glutathione (GSH), as well as the non-proteinaceous amino acid taurine. Vitamin B6 serves as a critical cofactor for these transsulfuration reactions, important part established in models of the calorie restriction (CR) benefits for longevity. Moreover, gut microbiota contributes to sulfur metabolism by producing hydrogen sulfide (H_2_S), a gasotransmitter arising from bacterial and yeast activity in the GIT, thereby linking Met turnover to microbiome-derived metabolic signaling [7,34]. These interconnected processes illustrate how nutrient availability, sulfur amino acid metabolism, and microbiome activity converge to influence hallmarks of health such as nutrient sensing, redox homeostasis, and host–microbiome communication (Figure 1).

SAMe also participates in polyamine biosynthesis, combining with ornithine (derived from arginine metabolism) to form putrescine, spermidine, and spermine—molecules essential for cellular growth, RNA stability, translation efficiency, and chromatin organization. Regeneration of 5′-methylthioadenosine (MTA) in the methionine salvage pathway similarly depends on zinc for the activity of 5-methylthioribulose-1-phosphate dehydratase (MTNB/APIP) [34,43,44].

Furthermore, the still unresolved paradox of the reported health and longevity benefits of Met restriction (MetR) in adult mammals is associated with dramatically increased Hyc levels. Deficiencies in Zn or in any of the B vitamins required for the methionine cycle may impair Hyc clearance, leading to elevated homocysteine, a widely used blood biomarker of disrupted 1C metabolism. Reduced taurine synthesis may also occur, contributing to metabolic dysfunction, elevated oxidative stress, and a range of human degenerative conditions. In some cases, these impairments may be ameliorated by targeted supplementation with B9, B12, B6, and zinc in combination, reflecting the cooperative roles of these micronutrients in maintaining metabolic integrity. Many population-controlled studies showed benefits of vitamin supplementation but usually without targeting their mutual synergy, and other interactions as these with eTMs, that may be determined through further research [7,43,44].

An important aspect of zinc–methionine interactions relate to differences in the initiation of protein synthesis between cytosolic and mitochondrial systems. In eukaryotes, cytosolic mRNA utilizes a single methionine codon, after which the zinc-dependent methionine aminopeptidase METAP1 removes most *N*-terminal Met residues to reduce vulnerability to oxidative damage. In contrast, mitochondrial mRNAs contain two codons for Met, increasing the abundance of Met residues and rendering mitochondrial proteins more susceptible to oxidation [34]. Under oxidative stress, Met undergoes spontaneous, reversible conversion to methionine-sulfoxide (MetO), functioning as a redox-sensitive PTMs. Further oxidation produces methionine-sulfone (MetO_2_), an irreversible state associated with impaired protein function due to ROS accumulation.

Reduction of MetO is mediated by methionine sulfoxide reductases, MsrA and MsrB1–3, which exhibit strict stereospecificity. MsrA, a ubiquitous selenoprotein, reduces the S-MetO epimer, whereas the MsrB family—three of which require zinc—reduces the R-MetO form. Notably, MsrB3 regeneration can be supported not only by glutaredoxin and thioredoxin systems but also by thioneins, thereby linking methionine repair to metallothionein-mediated redox buffering and zinc homeostasis [45,46].

Collectively, the direct and indirect intersections of zinc with methionine metabolism underscore zinc’s integration into intracellular oxidative stress pathways, PTMs, and epigenetic regulation, with distinct consequences in cytosolic and mitochondrial redox environments [32,33,34,43,44].

### 6.2. Zinc in Cysteine Metabolism and Redox-Dependent Protein Regulation

Cysteine (Cys), a conditionally essential sulfur-containing amino acid derived from Met metabolism, plays a central role in maintaining the three-dimensional structure of proteins, largely through the formation of disulfide bonds. These covalent linkages are critical for the stability, folding, and activity of a wide range of proteins with major physiological relevance. Beyond structural functions, Cys serves as an important redox-sensitive residue that can bind and release zinc, thereby modulating the conformation and activity of numerous zinc-associated proteins via ZPPIs and linking zinc availability to the cellular redox environment [2,4].

As described earlier, this redox-sensitive capability is particularly evident in iMTs, where transitions between multiple oxidation states of the metal-free thionein and zinc-bound metallothionein forms regulate both zinc sequestration and redox buffering. These dynamic interconversions enable iMTs to function as redox switches, coordinating Zn^2+^ release and sequestration according to cellular oxidative status and thereby influencing signaling pathways involved in stress responses, detoxification, and gene regulation [2,19,27,45,46,47].

Another well-defined example of zinc–cysteine coordination contributing to redox sensing occurs within intermediate filaments (IFs). Zinc binding to Cys residues stabilizes IF structure by protecting them from aberrant PTMs that would otherwise disrupt filament assembly, alter higher-order oligomerization, and impair cytoskeletal integrity. Loss of zinc-mediated stabilization under oxidative or inflammatory stress predisposes IF proteins to structural reorganization, potentially facilitating epithelial–mesenchymal transition (EMT), a process associated with tissue remodeling, fibrosis, wound repair, and pathological progression including cancer metastasis [47].

Together, these processes illustrate how Cys metabolism integrates with Zn-dependent redox regulation at structural and signaling levels. Through modulation of iMTs redox cycling, ZPPI-mediated enzyme control, and cytoskeletal stability, Zn and sulfur-containing amino acids jointly contribute to hallmarks of health including homeostatic resilience through processes of proteostasis, cellular identity, and controlled redox stress (Figure 1).

### 6.3. Klotho Protein, Longevity, the Metzincin Family, and Zinc–Vitamin D Interactions

The α-Klotho protein is widely recognized as a regulator of longevity. Originally identified through an unintended gene disruption, the resultant Klotho-deficient mouse exhibited a profound progeroid phenotype characterized by accelerated aging, reduced lifespan, and heightened susceptibility to stress. Conversely, α-Klotho overexpression in experimental animals extends healthy lifespan and enhances resilience to physiological perturbation. In mammals, including humans, circulating and membrane-bound α-Klotho levels decline progressively with chronological aging, whereas serum phosphate levels—under Klotho control—increase in parallel [10,48,49,50,51].

Membrane-bound α-Klotho functions as an obligate co-receptor for fibroblast growth factor 23 (FGF23), coordinating phosphate and vitamin D metabolism. β-Klotho, another family member, forms the co-receptor complexes for FGF19 and FGF21, thereby contributing to bile acid metabolism, energy expenditure, and integrated nutrient signaling [10,50]. Importantly, both α-Klotho expression and inducible metallothioneins (iMTs) can be upregulated by zinc, highlighting Zn’s multiple roles in stress defense, mineral homeostasis, and the potential to promote healthy aging [10,19,22].

Proteolytic cleavage generating the soluble form of α-Klotho is mediated predominantly by members of the zinc-dependent Metzincin superfamily, which encompasses matrix metalloproteinases (MMPs) and the a disintegrin and metalloprotease (ADAM) family. These metalloproteases are essential for tissue remodeling, barrier integrity, wound repair, and ectodomain shedding across multiple organ systems [49,50].

ADAM10 and ADAM17, functioning as α-secretases, release soluble α-Klotho into circulation. Soluble α-Klotho, which retains a glucuronidase-like domain, regulates turnover and trafficking of glycosylated membrane receptors and transporters, thereby exerting widespread endocrine, paracrine, and autocrine effects [10,49,50,51,52]. Linking ADAM metalloprotease activity and α-Klotho regulation as first instance of Zn dependencies is rarely acknowledged in the broader Klotho literature [8,10,48,49].

Experimental animal models demonstrate that circulating α-Klotho levels—an emerging biomarker of healthspan—can be increased by dietary phosphate restriction combined with zinc supplementation, with particularly notable effects in female mice [51]. Clinically, low-dose zinc supplementation has shown renal protective effects in pediatric chronic kidney disease (CKD), suggesting potential benefits of zinc interventions in CKD management and highlighting the need for further studies on dose and sex-specific responses [52]. Note, that common sex-specific pathophysiological responses in mammals, including humans, emerge not only from differences in circulating gonadal steroids but also from deeper, epigenetically imprinted layers of regulation [53]. These layers—including DNA methylation, chromatin architecture, and transcriptional responsiveness—are zinc dependent, pointing to zinc importance in mediating sex-specific metabolism, stress responses, and possible to aging trajectories, that warrant for further research in already mentioned synergy with many of essential nutrients.

Second instance of Zn interplay with α-Klotho is through obligatory activity of vitamin D3 (cholecalciferol). VD plays a role in the integrated regulation of phosphate, calcium, and magnesium metabolism to maintain skeletal integrity. However, its broader systemic and immunomodulatory functions involve complex interactions with zinc and α-Klotho that are often overlooked. A bidirectional feedback loop exists between α-Klotho signaling and VD status: α-Klotho suppresses excess vitamin D activation, while VD deficiency reduces Klotho expression. Together, this triad potentially influences not only mineral metabolism, but also oxidative stress responses, inflammation, and multiple hallmarks of health [10,30,54].

A recent review also highlighted population studies that showed associations between higher VD status and longer leukocyte telomere length and increased telomerase activity, consistent with delayed cellular senescence [43]. Considering the mandatory link between zinc and vitamin D activity—and their mutual dependence on vitamin A (retinoids) for optimal NRs activation—an integrated, combinatorial perspective is required. Expected benefits attributed to vitamin D require adequate zinc status, and conversely, many zinc-dependent processes may rely on vitamin D and vitamin A availability to reach full physiological and genomic potential [54].

### 6.4. Zinc, Vitamin D, Immunity, and the Gastrointestinal Tract (GIT)

Zinc exerts central immunoregulatory functions both independently and in cooperation with vitamins D and A. Beyond classical micronutrient–NR interactions, zinc participates in PTM-dependent signaling pathways within immune cells, particularly through the zinc-finger protein A20 (TNFAIP3). A20, together with its interacting partners—such as A20-binding inhibitor of NF-κB activation (ABIN)—acts as a negative regulator of NF-κB signaling by promoting ubiquitin-dependent termination of tumor necrosis factor (TNF)-induced inflammatory cascades. This zinc-dependent suppression of NF-κB activity provides a one of the mechanistic bases for the well-documented anti-inflammatory effects of zinc across multiple levels of research [8,54,55,56,57].

Importantly, A20 expression itself is modulated by vitamin D, positioning the zinc–VD axis as one of the determinants of immune responses. This cooperative regulation may partially explain the improved clinical outcomes reported for individuals receiving combined zinc and VD supplementation during acute respiratory infections, through the COVID-19 pandemic [43,54,58]. Maintaining immune homeostasis requires the coordinated activity of circulating and tissue-resident cells of the innate and adaptive systems. Disruption of these regulatory networks contributes to both autoimmunity (hyper-reactivity) and immunosenescence (hypo-reactivity) [54,57,58]. Given zinc’s pleiotropic actions—together with VD–driven transcriptional regulation—synergistic supplementation strategies warrant further mechanistic and clinical investigation, particularly in contexts of chronic inflammation, premature cellular senescence, and disorders of immune imbalance. Collectively, these interactions support a model in which zinc and VD form part of an integrated micronutrient circuit in hallmarks of health influencing telomere maintenance, inflammatory control, and systemic immune resilience (Figure 1).

Recent basic research through cellular and animal studies has also identified a significant, though predominantly indirect, role for zinc in the GIT through its interaction with the aryl hydrocarbon receptor (AhR). AhR senses diverse exogenous ligands, including nutritionally relevant phytochemicals such as resveratrol, and regulates gene expression of numerous transport systems via xenobiotic-responsive elements (XREs). Activation of AhR by metabolites of the essential amino acid tryptophan enhances zinc uptake across the intestinal epithelium by upregulating zinc transporter expression [8,11,55]. This coordinated response promotes the structural and functional integrity of tight junction barriers (TJBs), thereby supporting epithelial cohesion and potentially reducing susceptibility to the increased intestinal permeability characteristic of inflammatory bowel diseases (IBD). These findings are consistent with randomized controlled trials demonstrating benefits of the individual compounds, highlighting a direction for future studies on their combined synergistic effects to improve patient outcomes.

The emerging recognition of zinc as a critical regulator of epithelial barrier function highlights a translational gap between mechanistic evidence and population-level findings. Although observational studies consistently associate optimal zinc status with improved gastrointestinal and systemic health, controlled interventional trials—particularly those assessing combinations of zinc with vitamin D or other synergistic micronutrients—remain limited. Considering zinc’s contributions to epithelial renewal, cytoskeletal stability, and extracellular matrix interactions, a more integrated approach to micronutrient supplementation represents a promising direction for future clinical research.

## 7. Zinc in Molecular Mechanisms Underlying Diverse Medical Conditions

The clinical relevance of zinc first entered modern medical recognition through the seminal observations of Prasad in 1963, who identified severe dietary zinc deficiency and thereby established zinc as an essential trace element required for normal human growth, immune competence, neurodevelopment, and sexual maturation [57]. Since that time, extensive epidemiological, clinical, and mechanistic studies have demonstrated the role of zinc in molecular metabolism and in the pathobiology of a broad spectrum of genetic, metabolic, immune-mediated, degenerative, and infectious disorders as presented through previous chapters and continues below (Table 1) [8,9,10,11,12,13,14,15,16,57,58,59,60,61,62,63,64,65,66,67,68,69,70,71,72,73,74,75,76,77,78,79,80,81,82].

Although medical conditions represent pathological disruptions of physiological processes, they have simultaneously served as critical entry points for elucidating the biological circuitry underlying human health. Increasingly, evidence indicates that zinc participates directly or indirectly in many of these regulatory networks. In some cases, restoring or optimizing zinc status—either through dietary intake, targeted supplementation, or modulation of zinc transport—has been shown to alleviate molecular dysfunctions and support maintenance of homeostasis, a central hallmark of health.

The systemic organization of biological function can be conceptualized as comprising three interdependent components: (i) spatial compartmentalization, (ii) homeostatic maintenance, and (iii) stress responsiveness [5]. All three components depend on adequate availability of micronutrients, including zinc, other essential metals and minerals, fat- and water-soluble vitamins, and complementary phytonutrients derived from the diet. Each contributes to cellular integrity, proteostasis, redox balance, metabolic regulation, and adaptive responses to physiological stressors (Figure 1). Conversely, even partial deterioration of these micronutrient-dependent systems appears to characterize the decline associated with healthy aging and may underlie multiple chronic disease trajectories [5,6,17].

Zinc’s necessity is consistently observed across molecular, cellular, tissue, and organismal levels. The following sections highlight selected examples of molecular interconnections linking zinc biology with disease etiology or progression, drawing from conditions in which zinc plays a mechanistic role or where zinc supplementation has demonstrated preventive or therapeutic potential [8,9,10,11,12,13,14,15,16,53,57,58,59,60,61,62,63,64,65,66,67,68,69,70,71,72,73,74,75,76,77,78,79,80].

### 7.1. Zinc Benefits Across Medical Conditions in the Context of Maturation and Aging

Age-related macular degeneration (ARMD) represents one of the most prevalent causes of visual impairment in aging populations. Clinically, ARMD is classified into the neovascular (“wet”) and atrophic (“dry”) forms. Wet-ARMD is primarily characterized by pathological neovascularization and vascular leakage, for which anti-VEGF antibodies remain the standard of care. In contrast, the more common dry-ARMD—approximately four-fold more prevalent—presents with progressive accumulation of drusen, increased oxidative stress, impaired protein turnover, and chronic low-grade inflammation.

Clinical evidence from large-scale interventional trials demonstrates that the onset and progression of ARMD can be slightly delayed through multifactorial nutrient-based strategies incorporating zinc, antioxidant vitamins, and carotenoids such as lutein and zeaxanthin. These studies remain among the few examples of combined supplementation using essential and functional nutrients with documented efficacy in modulating degenerative disease outcomes. However, they have also been subject to ongoing debate, particularly regarding zinc speciation and dosage. Some of initial formulations containing 80 mg/day were later reduced to 45 mg/day following safety concerns [8,16].

The responsiveness of dry-ARMD to zinc—especially when combined with vitamins A, C, E, carotenoids, and omega-3 fatty acids—points to the requirement for adequate zinc availability in processes involved in oxidative stress control, proteostasis, complement regulation, and retinal epithelial integrity. These molecular processes are discussed extensively in earlier sections of this review and reflect zinc’s importance in maintaining cellular homeostasis throughout aging [8,16,55].

Zinc-dependent metabolic support is also observable in type 2 diabetes mellitus (T2D), a core component of the metabolic syndrome (Table 1). Pancreatic β-cells rely on zinc for insulin biosynthesis, storage, and secretion. Insulin is packaged into dense-core vesicles as a zinc-stabilized hexameric complex together with amylin. Perturbations in zinc homeostasis can therefore compromise proper insulin maturation, vesicular loading, and controlled release. Furthermore, inadequate zinc availability has been implicated in impaired lysosomal acidification and autophagy–lysosomal pathway (EALP) function, leading to accumulation of protein aggregates, including amylin.

Beyond pancreatic β-cell biology, whole-body glucose sensing and metabolic homeostasis rely on multiple zinc-dependent enzymes, signaling molecules, and transcriptional regulators. In the context of obesity-associated metabolic syndrome, zinc-α2-glycoprotein (ZAG) is of particular relevance. ZAG, structurally related to major histocompatibility complex class I (MHC I) molecules, acts as a lipid-mobilizing adipokine that enhances lipolysis, supports energy expenditure, and improves insulin sensitivity. Reduced ZAG activity, potentially associated with zinc insufficiency, may contribute to impaired adipose tissue metabolism and insulin resistance, highlighting the need for further multi-layered studies [8,13,41,58].

Reproductive function in both sexes provides another domain in which zinc plays a significant physiological and molecular role (Table 1). Zinc supports gametogenesis, meiotic maturation, genomic stability, chromatin remodeling, and oxidative protection in germ cells across the reproductive lifespan. At fertilization, as an example, a rapid and tightly coordinated zinc flux—commonly termed the “zinc spark”—occurs upon oocyte activation. This process is initiated by contact with functional spermatozoa that have completed capacitation following dilution of zinc-rich prostatic fluid. Reduction in extracellular zinc allows sperm to acquire hypermotility and enables full activation of mitochondrial ATP production necessary for fertilization competence [8,37,53].

### 7.2. Zinc Dysregulation in PAMI: A Peculiar Autoinflammatory Disorder

The exceptionally rare genetic disorder initially described as hyperzincemia–hypercalprotectinemia (Hz/Hc) syndrome and later reclassified as PSTPIP1-associated myeloid-related proteinemia inflammatory (PAMI) syndrome is caused by two pathogenic mutations in the PSTPIP1 gene. These variants alter the charge distribution at the protein–protein interaction interface, resulting in aberrant signaling within innate immune pathways [60,61].

PAMI is characterized by a paradoxical combination of markedly elevated circulating zinc concentrations and concurrent functional zinc deficiency at the tissue level (Table 1). This biochemical contradiction arises from the unregulated release and persistent elevation of alarmins of the S100 family, particularly the proinflammatory heterodimer myeloid-related protein 8/14 (MRP8/14; also known as S100A8/S100A9). Calprotectin, the MRP8/14 complex, exhibits high-affinity binding to multiple divalent cations—including Ca^2+^, Zn^2+^, Mn^2+^, and Fe^2+^—with substantially increased affinity upon heterodimerization or tetramerization. Excessive calprotectin in the circulation thus sequesters zinc, trapping it in non-bioavailable complexes and depriving tissues of exchangeable zinc pools despite the apparent hyperzincemia [60]. This feature distinguishes PAMI from other PSTPIP1-associated inflammatory diseases (PAID), which are linked to alternative PSTPIP1 mutations but do not show the same extreme elevations of MRP8/14 or zinc.

Clinically, PAMI manifests with chronic systemic inflammation, neutropenia that persists despite immunosuppressive treatment, anemia, cutaneous involvement, osteoarticular inflammation, growth impairment, and organomegaly—particularly hepatosplenomegaly. Some manifestations respond partially to immunosuppressive therapy, while neutropenia remains refractory. The overall clinical picture reflects sustained innate immune activation and profound dysregulation of metal homeostasis.

PSTPIP1 itself is a multifunctional cytoskeletal-associated adaptor protein expressed predominantly in hematopoietic lineages. It participates in membrane anchoring, protein docking, and regulation of turnover with activation of inflammatory mediators. Mutations leading to PAMI alter PSTPIP1’s interactions with another Zn containing, actin-associated protein, pyrin a component of inflammasome signaling, thereby amplifying inflammatory cascades [60].

Physiologically, transient increases in serum zinc form part of the innate immune defense, promoting antimicrobial activity while allowing calprotectin to sequester zinc and inhibit bacterial and fungal proliferation. In PAMI, however, this protective mechanism becomes chronically activated. Persistent release of MRP8/14 sustains a pathological loop wherein zinc is continuously sequestered in plasma, leaving tissues—particularly immune cells—functionally zinc-deficient despite elevated serum zinc levels [8,58,60].

### 7.3. Zinc Transporters in Health and Disease

Acrodermatitis enteropathica (AE) is a rare autosomal recessive disorder characterized clinically by severe periorificial and acral dermatitis, alopecia, diarrhea and failure to thrive. Molecular analyses have established that AE results from loss-of-function mutations in the intestinal zinc importer ZIP4 (SLC39A4), leading to markedly impaired dietary zinc absorption [57,59]. AE represents one of the earliest examples of a human disease successfully treated with oral zinc supplementation. Upregulation of compensatory transporters, such as divalent metal transporter 1 (DMT1/SLC11A2), can contribute to partial zinc uptake across the apical enterocyte membrane and is further stimulated by supplemental zinc intake.

A related but temporary condition, transient neonatal zinc deficiency (TNZD), arises in infants breastfed by mothers who are heterozygous carriers of SLC30A2 (ZnT2) mutations. In this scenario, maternal milk fails to provide adequate zinc, resulting in an AE-like presentation in the infant despite normal intestinal transport capacity [59]. TNZD is responsive to zinc supplementation, underscoring the role of ZnT2 in mammary gland zinc transport and highlighting the importance of adequate maternal zinc status not only during pregnancy but also throughout the lactation period.

More complex autosomal recessive syndromes result from intracellular zinc mislocalization due to mutations affecting specific zinc transporters. Spondylocheirodysplastic Ehlers–Danlos syndrome (SCD-EDS) is caused by mutations in ZIP13 (SLC39A13), which impair zinc export from the Golgi apparatus into the cytosol. ZIP13 is required for proper activation of bone morphogenetic protein (BMP) and transforming growth factor-β (TGF-β) signaling pathways. Defective cytosolic zinc availability in ZIP13 deficiency leads to impaired connective tissue development, characteristic musculoskeletal abnormalities, and long-term consequences for stature, joint integrity, and quality of life.

Another emerging transporter-related disorder is Birk–Landau–Perez syndrome (BILAPES), resulting from mutations in ZnT9 (SLC30A9). This condition manifests as a cerebro-renal syndrome with neurodevelopmental impairment and progressive renal dysfunction. The precise mechanisms remain under investigation and may involve endoplasmic reticulum (ER) stress, mitochondrial ETC dysfunction, and/or a gain-of-function effect that alters zinc handling within organelles. Mutations inside the putative transition pore domain of ZnT9 have been proposed to affect β-catenin/Wnt signaling, contributing to the complex phenotype [59,62].

Collectively, these disorders highlight the importance of Zn transporters in maintaining appropriate intracellular Zn distribution and supporting tissue-specific physiological functions (Table 1). Beyond rare genetic diseases, deregulated expression of ZIP and ZnT transporters is recognized in numerous cancers, metabolic disturbances, and inflammatory conditions. Their roles in cellular signaling, organelle function, and ZPPIs are highly context- and tissue-dependent, underscoring the broader significance of disruptions in Zn transporter biology for human health and disease, which remains an active and intensively studied filed of research [8,32].

### 7.4. Zinc in Disorders of Iron Metabolism

Several inherited disorders of metal metabolism exhibit partial responsiveness to Zn supplementation. These include thalassemia and hereditary hemochromatosis, both of which are characterized by systemic Fe overload [59]. In these conditions, Zn may mitigate secondary complications—such as oxidative stress, altered erythropoiesis, inflammation, and endocrine dysfunction—through basic mechanisms discussed in earlier sections, that also warrant further layers of investigations.

Zinc deficiency is commonly associated with iron-deficiency anemias, particularly in contexts where erythropoietic activity is altered. The high turnover rate of erythrocyte production in mammalian bone marrow depends not only on Fe availability but also on Zn-dependent enzymes and epigenetic regulators that govern chromatin condensation and nuclear extrusion during late erythroblast differentiation. Thus, combined deficiencies of Fe, Zn, and vitamins B6, B9, and B12 can disrupt erythropoiesis, yet this multifactorial interplay has only recently gained broader recognition in clinical hematology [31].

Currently, clear treatment guidelines are well established only for specific etiologies—such as iron-deficient anemia, sideroblastic anemia (vitamin B6-responsive forms) and pernicious anemia (vitamin B12 deficiency). However, standardized clinical investigations examining combined micronutrient supplementation across different age groups, sexes, and physiological states remain limited.

Existing studies evaluating Zn supplementation in anemic conditions report heterogeneous outcomes, influenced by numerous variables including age, zinc speciation and bioavailability, baseline nutritional status, inflammatory state, and specific hematological parameters assessed [8,9,30,31,53,59]. As such, although Zn is recognized as an important modulator of erythropoiesis and Fe homeostasis, further controlled trials are required to clarify dosage, timing, and therapeutic efficacy across diverse clinical populations.

### 7.5. Zinc Necessity in Lysosome Disorders Through Lysosome Functioning and Turnover

Lysosomal storage disorders (LSDs) comprise a heterogeneous group of approximately 70 rare genetic diseases characterized by the lysosomal accumulation of undegraded or partially degraded substrates. This hallmark phenotype typically results from mutations that impair the function, trafficking, or stability of lysosomal hydrolases. However, substrate accumulation can also arise from deficient lysosomal transport mechanisms, leading to impaired substrate efflux despite intact enzymatic activity. Although detailed evaluation of adjunctive therapies for LSDs lies beyond the scope of the present work, emerging evidence suggests that Zn supplementation may offer supportive benefits through multiple established mechanistic pathways illustrated below.

Recent basic biology studies indicate that zinc enhances lysosomal acidification by promoting the assembly and stability of the vacuolar H^+^-ATPase (V-ATPase) multimeric complex, thereby improving substrate degradation efficiency within the lysosomal compartment [63,64]. Given that optimal V-ATPase functionality is essential for maintaining the low pH required for enzymatic activity, Zn-dependent stabilization of this machinery may partially compensate for the enzymatic deficits typically observed in LSDs and potentially prevent continuous accumulation of undegraded material.

Additionally, Zn plays a role in autophagy and endo-lysosomal turnover, EALP. A key regulatory element in this context is the family of palmitoyl acyltransferases (PATs), also designated as ZDHHC enzymes. These enzymes catalyze cysteine palmitoylation—a major PTMs required for the membrane association, trafficking, and function of numerous autophagosomal proteins [65]. The conserved Asp-His-His-Cys (DHHC) catalytic motif, together with essential zinc finger (ZNF) structural domains, underscores the dependency of this enzyme family (ZDHHC1–24, excluding ZDHHC10) on zinc for correct folding and enzymatic activity [65,66]. The rapidly expanding recognition of cysteine palmitoylation as a PTM regulator of membrane protein dynamics further underlines zinc’s structural and catalytic contributions, established on mechanistic level of evidence.

These zinc-dependent mechanisms are probably relevant in several neurodegenerative diseases (NDDs), where impaired autophagy and lysosomal dysfunction are prominent pathogenic features. Zinc’s antioxidative properties, together with its involvement in mitophagy and ferritinophagy, place zinc as a possible potential long-term stabilizing factor in lysosomal homeostasis and cellular stress responses. Although zinc supplementation will not resolve any forms of LSDs, though many synergy circuits, it may provide supportive benefits in mitigating some of systemic consequences in conditions where lysosomal function, turnover, and redox balance are compromised, that could be direction for future studies.

Moreover, as established at the basic molecular level, Zn, which is integral to NRs, mediates regulation of lysosomal biogenesis and potential exocytotic removal of old lysosome content. Activation of peroxisome proliferator-activated receptor-α (PPARα) has been shown to enhance expression of transcription factor EB (TFEB), the master regulator of lysosomal biogenesis gene networks. TFEB controls more than 400 genes involved in lysosomal formation, autophagy, metabolism, and vesicular trafficking [8,22,67]. As PPARα function depends on zinc-binding domains for structural integrity and DNA interaction, zinc availability is required for maintaining lysosomal biogenesis and cellular proteostasis.

Overall, despite incomplete elucidation of all mechanistic pathways, accumulated evidence underscores zinc’s involvement in lysosomal acidification, autophagic flux, post-translational regulation, and transcriptional control. These interconnected roles highlight zinc as an important micronutrient in cellular defense and adaptation, with potential relevance in the context of LSDs and other conditions characterized by impaired lysosomal function that deserve further levels of studies for patients benefit.

### 7.6. Zinc Role in Neurodegenerative Diseases

The possible clinical utility of zinc supplementation in neurological and neurodevelopmental conditions has been articulated extensively by Hoogenraad, who outlined the historical use of zinc in epilepsy management until the late 20th century and, supported by accumulating mechanistic evidence, subsequently advocated its therapeutic prospects also in multiple neurodegenerative diseases (NDDs) [68]. The cellular and molecular processes discussed in previous sections—including ZPPIs, redox-regulated zinc release, EALP function, and zinc-dependent proteostasis—converge to support this emerging conceptual framework.

Alzheimer’s disease (AD) exhibits multiple pathological hallmarks, notably extracellular β-amyloid plaques. Zinc plays diverse roles in the regulation of amyloid precursor protein (APP) processing. Members of the Metzincin family, particularly ADAM10 and ADAM17, compete with β- and γ-secretase complexes for APP cleavage, thereby determining the balance between non-amyloidogenic P3 fragment production and amyloidogenic fragment formation [8,50,68]. Dysregulated zinc homeostasis may shift this balance toward pathogenic pathways.

Furthermore, numerous NDDs—including Alzheimer’s disease (AD), amyotrophic lateral sclerosis (ALS), Parkinson’s disease (PD), Huntington’s disease (HD), and prion disorders—are characterized by intracellular protein aggregates that may accumulate, in part, due to impaired lysosomal acidification and deficient autophagy–lysosomal pathway (EALP) activity. Zinc availability is critical for maintaining the structural integrity and catalytic activity of V-ATPase, autophagy regulators, and multiple proteases required for adequate degradation of misfolded proteins. Consequently, zinc deficiency or mislocalization can impair neuronal proteostasis, contributing to aggregate accumulation and progressive neuronal dysfunction.

Dietary factors further modulate these risks. Higher intake of zinc, vitamin B12, and PUFAs has been associated with reduced AD incidence in population studies [50]. Lipofuscin accumulation—a hallmark of cellular aging—also reflects chronic inefficiency in lysosomal degradation, incorporating numerous eTMs. Excessive or misplaced PTMs, including dysregulated phosphorylation and palmitoylation, further exacerbate aggregate formation and involve zinc-dependent enzymatic systems [8,22,45,55,63,64,65,66,67,68,69,70,71,72,73].

The interaction between zinc status and female-specific physiology has gained particular attention. Population studies indicate that the menopausal transition is closely linked to increased AD risk, contributing to the observed female predominance. During this period, declining ovarian estrogen levels are accompanied by increased estrogen receptor-α (ERα) expression in the brain. Adequate zinc availability is essential for optimal ERα structure and function, reinforcing the need for precise zinc localization during this transition [14,53,68,69,70,71,72]. Zinc deficiency or misplacement is consistent with both metal dyshomeostasis and metabolic hypotheses of AD pathogenesis. Broader menopausal symptoms—from vasomotor instability to osteoporosis and age-related chronic conditions—should therefore be considered through the integrated lens of zinc-dependent NR signaling involving estrogen, vitamin D, magnesium, and other essential minerals, as outlined above.

Emerging evidence further highlights the potential of zinc-containing bioactive complexes. Zinc–carnosine, named polaprezinc (β-alanyl-l-histidine), a naturally occurring dipeptide derivative, exhibits anti-aggregatory effects and can inhibit protein oligomerization across multiple NDD-related pathways [73]. Beyond neuroprotection, zinc–carnosine has demonstrated beneficial effects in bone and cartilage regeneration, as well as in maintaining α-crystallin stability in the eye lens, where it may delay or reduce cataract formation [30,73]. These properties need further research to confirm the role of zinc–carnosine and related compounds as promising candidates for preventive and therapeutic strategies across a spectrum of age-related degenerative conditions.

### 7.7. Zinc Assistance in Wilson’s Disease

Wilson’s disease is an autosomal recessive hereditary disorder of copper metabolism caused by loss-of-function mutations in ATP7B, a copper-transporting P-type ATPase required for copper excretion from hepatocytes into bile. Defective ATP7B function results in progressive hepatic copper accumulation, followed by systemic redistribution to extrahepatic tissues—most prominently the brain and eyes—ultimately leading to hepatic cirrhosis, neuropsychiatric manifestations, and ophthalmologic signs such as Kayser–Fleischer rings [8,9,59,74].

Zinc therapy represents a highly effective and well-tolerated approach for Wilson’s disease and has been championed extensively by Hoogenraad, who demonstrated zinc’s low toxicity profile compared with traditional chelation regimens. Historically, D-penicillamine served as the principal therapeutic option through its chelating activity, promoting urinary copper excretion. However, its use is frequently limited by adverse events ranging from gastrointestinal intolerance to severe immunological and neurological reactions. Notably, transient elevation of circulating copper–chelator complexes can cross the blood–brain barrier, potentially exacerbating neurological injury in susceptible patients [59,74].

Contemporary treatment guidelines recognize zinc as a first-line therapy, either as monotherapy—particularly in presymptomatic or maintenance phases—or in combination with chelators during initial de-coppering. High therapeutic doses, typically up to 6 mg Zn/kg body mass/day, administered as zinc acetate or other bioavailable salts, effectively reduce systemic copper burden with minimal side effects, most commonly transient gastrointestinal discomfort [59,74,75].

The principal mechanism underlying zinc’s effectiveness involves its ability to induce intestinal metallothioneins (iMTs) in GIT epithelial cells. Metallothioneins exhibit a markedly higher binding affinity for copper than for zinc; thus, zinc-induced iMTs sequester dietary copper within enterocytes, which are subsequently sloughed during normal epithelial turnover, thereby reducing net copper absorption. A similar mechanism occurs in hepatic and other systemic tissues, where elevated zinc concentrations stimulate iMTs synthesis, promoting intracellular copper sequestration and reducing its bioavailable pool. This dual effect—limiting copper uptake and enhancing intracellular copper binding—accounts for zinc’s clinical efficacy and its long-term safety in Wilson’s disease management.

Given its favorable risk–benefit profile, ability to reduce copper absorption, and compatibility with lifelong therapy, zinc supplementation has become an essential component of evidence-based Wilson’s disease treatment strategies [59,74,75].

### 7.8. Considerations Regarding Zinc Toxicity

The medicinal use of zinc dates back to ancient civilizations, with early preparations derived from mineral ores such as sphalerite and wurtzite (ZnS), and calamine (primarily ZnCO_3_), widely applied for dermatologic conditions, wound healing, and ocular irritation as early as 1500 B.C [2,19,73]. Despite this long history of use, concerns about potential zinc toxicity periodically re-emerge and must be addressed within an evidence-based framework. Most reports suggesting toxicity originate from either experimental animal studies employing extreme supraphysiological doses or isolated case reports involving inappropriate chronic exposure, such as excessive denture cream use or misguided self-administration of very high supplemental doses unaligned with clinical indications [76].

For humans, zinc doses demonstrating therapeutic benefit across diverse physiological and pathological conditions generally range from the recommended dietary allowance (RDA) for healthy individuals to moderately elevated intakes adjusted for age and sex and guided by clinical context [16,76]. Evidence discussed in the preceding sections supports the safety of zinc supplementation at approximately 1 mg/kg body mass/day, a dose generally associated with minimal and typically transient gastrointestinal side effects, and after establishing current status, in concordance with clinical therapy and through professional evaluation, probable appropriate for conditions of increased physiological demand, including aging, chronic metabolic stress and some of disorders discussed through this work.

When zinc intake rises above this threshold, such as in the management of Wilson’s disease, routine monitoring of copper status becomes prudent. Zinc’s mechanism of action in these settings involves metallothionein induction and subsequent copper sequestration; thus, appropriately monitored Zn and periodic assessment that ensures copper levels remain within the desired range, aiming to identify the rare cases in which minimal copper supplementation (≥0.9 mg/day) may be required to maintain systemic balance.

Overall, contemporary clinical and biochemical evidence indicates that therapeutic zinc supplementation within established dosing ranges is safe, well-tolerated, and effective in many cases. Success of Zn particularly when administered alongside other essential nutrients required for coordinated metabolic function certainly warrants more studies of appropriate and rational combinations.

### 7.9. Insight into Kleefstra Syndrome (KSS), Autism Spectrum Disorder (ASD) and the Role of Zinc

The rare neurodevelopmental disorder Kleefstra syndrome (KSS) has been elucidated through genetic and epigenomic analyses as a condition in which pathogenic variants converge on chromatin-modifying mechanisms. Clinically, KSS presents with intellectual disability (ID), hypotonia, and features of autism spectrum disorder (ASD) (Table 1) [76].

Molecular studies identified KSS as a 9q34 microdeletion resulting from haploinsufficiency of euchromatin histone lysine N-methyltransferase 1 (EHMT1/GLP), a repressive histone methyltransferase containing a zinc-binding SET domain [33,77]. Loss of EHMT1 prevents the formation of the functional GLP–G9a (EHMT1–EHMT2) heterodimer, reducing H3K9 mono- and dimethylation. This derepression leads to enhanced expression of neuronal genes, including the NR1 subunit of the N-methyl-D-aspartate receptor (NMDAR), a process strongly implicated in ASD-related synaptic dysfunction [18,77,78,79,80,81].

In glutamatergic synapses, zinc is co-released with glutamate and interacts with the NMDAR complex. Depending on receptor subunit composition, Zn^2+^ acts as a physiological inhibitor at the NR2A site, whereas Mg^2+^ regulates voltage-dependent blockade of the channel pore [8,17,18]. Disruption of these tightly regulated zinc-dependent synaptic mechanisms may exacerbate abnormal Ca^2+^ influx and downstream excitotoxic signaling.

Beyond EHMT1, at least four additional mutated genes linked to KSS encode proteins mediating histone PTMs (writing, reading, or erasing), highlighting the intricate epigenetic architecture of ASD susceptibility [77,78,79,80,81]. More recently, mutations in NR1I3—encoding the constitutive androstane receptor (CAR)—have been identified in KSS. Like the VD receptor (VDR), CAR requires dimerization with retinoid X receptor (RXR), a zinc-dependent nuclear receptor, thereby extending the mechanistic intersections between zinc, nuclear signaling, and transcriptional regulation [40].

Although ASD has established genetic underpinnings, its increasing prevalence—exceeding 1% and disproportionately affecting males—strongly suggests environmental, nutritional, and occupational influences. Vitamin D deficiency is consistently reported in ASD cohorts and is also widespread among women of reproductive age. Reduced sunlight exposure, indoor work environments, and decreased activity of VD–activating enzymes contribute to low 25(OH)D levels. In this context, maternal zinc insufficiency may further impair VD signaling. Notably, estrogen can partially compensate for VD deficiency, potentially contributing to sex-specific vulnerability in ASD development [53,78]. Furthermore, the VD response element (VDRE) in the promoter of glutamate decarboxylase (GAD) links VD status directly to GABA synthesis and influence on the inhibitory counterbalance required at glutamatergic synapses [54].

Another key component of ASD neurobiology is SHANK3, a postsynaptic density (PSD) protein essential for glutamatergic synapse integrity. SHANK3 mutations cause a spectrum of ASD phenotypes, including Phelan-McDermid syndrome. Zinc stabilizes SHANK3 structure and supports its scaffolding interactions with NMDARs and other postsynaptic components. Experimental models demonstrate that zinc supplementation can rescue defective synaptic architecture and normalize glutamatergic signaling in SHANK3 deficiency. This effect is consistent with zinc’s capacity to modulate NMDAR activity and reduce excessive Ca^2+^-dependent currents, thereby regulating long-term potentiation (LTP) and synaptic plasticity [77,78,79,80,81].

### 7.10. Aging and Zinc

Aging is a major risk factor for the development of most chronic, degenerative, and metabolic disorders, with clear sex-specific differences in incidence and progression [14,53,82]. Numerous observational and mechanistic studies consistently demonstrate that advancing age is accompanied by a measurable decline in systemic and cellular Zn status, which is implicated in metabolic dysregulation, cardiovascular disease, immune system deterioration, neurodegenerative disorders, and several malignancies [7,8,10,16,17,26,29,34,45,51].

The present review emphasizes biological processes that enable maintenance of functional integrity across hallmarks of health organizational levels and highlights conditions that remain modifiable through the coordinated action of essential and functional nutrients, including Zn [5,83]. Across multiple theoretical frameworks of aging, Zn emerges as a micronutrient required to maintain homeostasis and attenuate functional decline. Within the oxidative stress theory of aging, Zn contributes to the control of ROS production, supports antioxidant defenses, and protects endothelial and vascular integrity. In immunosenescence, suboptimal Zn availability impairs both innate and adaptive immune responses, reduces thymic function, and affects cytokine balance. In the context of antagonistic pleiotropy and nutrient-sensing pathways, Zn modulates mTORC1-related signaling cascades that influence cellular growth, metabolism, and longevity [82].

The most recent “information theory of aging” proposes that age-related decline in cell identity and functional coherence results from progressive loss of epigenetic fidelity, particularly in chromatin structure, histone modifications, and DNA methylation maintenance [84]. As discussed throughout this review, Zn is indispensable for the function of numerous enzymes and regulatory proteins that establish, interpret, and preserve epigenetic marks, including Zn-dependent transcription factors, DNA/RNA-binding proteins, deacetylases, methylases, and chromatin remodeling complexes. Thus, maintaining adequate Zn status may be required not only for classical homeostatic processes but also for sustaining the stability of epigenetic information across the lifespan.

## 8. Conclusions and Perspective

The involvement of essential transition metals (eTMs) in the structural and functional organization of cells is deeply rooted in evolutionary biology. Among these, zinc emerges as an essential trace element with uniquely broad biological relevance, encompassing structural, catalytic, regulatory, and signaling functions. Its biologically active forms—including tightly bound structural and catalytic zinc within proteins, transiently exchangeable intracellular zinc pools, and dynamic zinc signaling events—collectively support the molecular processes underlying the hallmarks of health.

The concept of a zinc functional interactome offers an integrative framework in which zinc functions as a coordinating node across the hallmarks of health. Through zinc-dependent molecular networks, adaptable cellular targets are linked to appropriate physiological responses, including epigenetic regulation, transcriptional control, enzymatic activity, immune competence, redox homeostasis, endocrine balance, and cellular renewal. In this context, zinc contributes to the maintenance of health not as an isolated factor, but as an integral component of interconnected regulatory and metabolic systems that is also exemplified through various presented disorders.

Health is sustained through the cooperative activity of biomolecules operating within dynamic biological cycles. Vitamins, steroid hormones, nuclear receptors, and other functional nutrients frequently rely on synergistic or obligate zinc-dependent interactions to exert their biological effects. Through these interactions, zinc supports multiple hallmarks of health simultaneously, contributing to systemic regulation, and has potential to mitigate functional decline associated with aging and various diseases, which deserves future research for establishing recommendable protocols.

Growing evidence indicates that combined investigation of zinc with other essential nutrients may advance strategies aimed at preserving or restoring the hallmarks of health via shared molecular pathways that maintain homeostasis. A prominent, though not yet fully elucidated, example is the interaction between zinc status and vitamin D–dependent signaling. Their combined activity extends beyond skeletal integrity and appears relevant to immune competence and the regulated expression of longevity-associated genes such as α-Klotho. Additionally, emerging research suggests roles for vitamin D–related pathways in telomerase regulation and telomere length stability—processes central to health maintenance and aging trajectories—that may be influenced by adequate zinc availability.

In summary, this conceptual and exploratory framework positions zinc as a core element of a functional interactome that underpins the hallmarks of health and supports biological resilience. Although this review provides a selective rather than exhaustive mapping of zinc-dependent interactions, it underscores zinc’s relevance as an adjunctive nutritional factor in maintaining health and modulating functional outcomes across both common and rare disorders. Moreover, this hallmarks-of-health–centered perspective may inform future efforts to model comprehensive cellular interactomes, including computational and AI-assisted approaches, in which zinc-dependent regulatory architectures serve as an instructive and scalable scaffold.

## Figures and Tables

**Table 1:** Overview of disorders with mechanistic involvement of zinc metabolism or documented benefit from zinc supplementation, as summarized in recent reviews [8,9,10,11,12,13,14,15,16,57,58,59,60,61,62,63,64,65,66,67,68,69,70,71,72,73,74,75,76,77,78,79,80,81,82].

Disorder Category	Condition	Key Mechanisms/Molecular Targets	Evidence for Zinc Support
Neurodevelopmental	Autism Spectrum Disorder	NMDAR &	Yes/Nc
	Kleefstra Syndrome	ECMT1/GLP &	Na
	Shankopathies	SHANK3 &	Yes/Nc
Neurodegenerative	Alzheimer’s Disease	Amyloid beta, TAU &	Yes/Nc
	Parkinson’s Disease	alpha-Synuclein &	Yes/Nc
	Huntington’s Disease	polyQ-HTT, SP1 &	Yes/Nc
	Amyotrophic Lateral Sclerosis	SOD1 &	Na
	Prion Disease	Prion Protein &	Na
Zinc Transport Defects	Acrodermatitis Enteropathica	ZNT4	Yes
	Transient Neonatal Zinc Deficiency	ZNT2	Yes
	Spondylocheirodysplastic Ehlers-Danlos Syndrome	ZIP13	Na
	Birk-Landau-Perez Syndrome, BILAPES	ZNT9	Na
Metabolic	Metabolic Syndrome	Various Causes	Yes
Miscellaneous	Pernicious Anemia	RBC Maturation	Yes/Nc
	Lysosomal Storage Disorder	Various Proteins	Yes/Nc
	ARMD	Various Proteins	Yes/Nc
Immune-Related	Pami Syndrome	PSTPIP1	Na
Infectious	COVID-19 &	Sars Virus	Yes
Genetic/Metal Overload	Wilson’s Disease	ATP7B, Cu	Yes
	Thalassemia	Fe Overload	Yes/Nc
	Hereditary Hemochromatosis	Various Proteins	Yes/Nc
Reproductive	Male Spermatogenesis & Fertility	Various Causes	Yes
	Female Fertility &	Various Causes	Yes

Legend: & = Multifactorial Involvement; Na = Data Not Available; Nc = Data Not Conclusive.

## Data Availability

No new data were created or analyzed in this study. Data sharing is not applicable to this article.

## Abbreviations

The following abbreviations are used in this manuscript:

AD	Alzheimer’s Disease
ADAM	A Disintegrin And Metalloprotease
ADMETox	Absorption, Distribution, Metabolism, Excretion and Toxicity
AE	Acrodermatitis Enteropathica
AI	Artificial Intelligence
ALS	Amyotrophic Lateral Sclerose
ARMD	Age Related Macular Degeneration
ASD	Autism Spectrum Disorder
CAs	Carbonic anhydrases
EALP	Endosomal–AutophagoLysosomal Pathway
ER	Endoplasmic Reticulum
ETC	mitochondrial Electron Transport Chain
eTMs	essential Transition Metals
EHMT	euchromatin histone lysine *N*-methyltransferases
GIT	GastroIntestinal Tract
HD	Huntington’s Disease
Hz/Hc	Hyperzincemia/Hypercalprotectinemia syndrome
IMS	mitochondrial InterMembrane Space
iMTs	inducible MetalloThioneins
KSS	Kleefstra syndrome
LSDs	Lysosomal Storage Disorders
NEWS	Nutrition, Exercise, Weight control, Sleep & Social interaction vs. Stress
NDD	NeuroDegenerative Diseases
NMDAR	*N*-methyl-d-aspartate receptor
NRs	nuclear receptors
PAMI syndrome	PSTPIP1 (Proline—Serine—Threonine Phosphatase—Interactive Protein 1)—Associated Myeloid—related proteinemia Inflammatory syndrome
PARPs	Poly (ADP-Ribose)-Polymerases
PD	Parkinson’s Disease
PPARs	Peroxisome Proliferator-Activated Receptors
PTMs	PostTranslational Modifications
PUFA	PolyUnsaturated Fatty Acids
ROS	Reactive Oxygen Species
RXR	Retionoid nuclear Receptor
SOD	Superoxide Dismutase
TCA cycle	Three Carboxylic Acid cycle in mitochondria
TNFAIP3/A20	TNF-α—Induced Protein 3/A20
Ub	ubiquitin
UPS	ubiquitin proteasome system
VD	Vitamin D3, calcitriol
ZNF	zinc finger motif in proteins
ZTF	zinc finger transcription factors
ZPPIs	Zn^2+^-involved Protein–Protein Interactions
ZIP/SLC39A	Zinc related transporters/Iron related transporters (Zrt/Irt) Protein/solute carrier family 39A
ZnT/SLC30A	ZnT/SLC30A, Zn Transporter family/solute carrier family 30A
